# Childhood white matter organization predicts adolescent internalizing problems among youth with and without ADHD

**DOI:** 10.1016/j.nicl.2026.104053

**Published:** 2026-09-01

**Authors:** C. Hyde, I. Fuelscher, K.S. Rosch, K.E. Seymour, D. Crocetti, T. Silk, M. Singh, S.H. Mostofsky

**Affiliations:** a School of Psychology, Deakin University, Burwood, Australia; b Center for Neurodevelopmental and Imaging Research, Kennedy Krieger Institute, Baltimore, MD, USA; c Department of Neuropsychology, Kennedy Krieger Institute, Baltimore, MD, USA; d Department of Psychiatry and Behavioral Sciences, Johns Hopkins University School of Medicine, Baltimore, MD, USA; e National Institute on Drug Abuse, Department of Health and Human Services, Rockville, MD, USA; f Murdoch Children's Research Institute, Melbourne, Australia; g Department of Neurology, Johns Hopkins University School of Medicine, Baltimore, MD, USA

**Keywords:** ADHD, Internalizing problems, Motor, Longitudinal, White matter, Fixel based analysis

## Abstract

This study investigated whether childhood white matter organization in those with and without ADHD predicted the emergence of internalizing problems in adolescence. Further, we aimed to determine whether (i) this longitudinal effect was mediated by motor skill level, and/or (ii) white matter organization in children with ADHD mediated the expected relationship between motor skill level and internalizing problems in adolescence. Participants were 40 children with ADHD and 36 typically developing (TD) controls aged between 8 and 13 years, who were followed-up during adolescence (12–18 years). All underwent diffusion MRI at Time 1, and assessment of motor competence using the Movement ABC-2 (MABC-2). At Time 2, internalizing problems (i.e., ‘depression’, ‘anxiety’, and ‘somatization’) were measured using the parent- and self-report Behavior Assessment System for Children (BASC). Following pre-processing of diffusion MRI scans, fixel based analysis was conducted and fibre cross-section (FC) extracted. TractSeg was then used to delineate the superior longitudinal fasciculus (SLF), inferior longitudinal fasciculus (ILF) and the uncinate fasciculus (UC). Results showed that childhood FC within the UF was a significant predictor of parent and child-rated depression scores in adolescence. Further, childhood FC of the bilaterial ILF was a significant predictor of parent report depression scores in adolescence. These effects were stable for those with and without ADHD, and no group differences in FC were observed in those white matter regions found to be associated with adolescent depressive symptoms. We failed to find evidence that motor ability mediated the relationship between white matter organization of the UF of ILF and depressive symptoms. When childhood motor ability was considered as a predictor of adolescent depressive scores (an effect that was significant for parent reported effects), this effect was mediated by childhood FC of the UF. This work suggests that low motor skill in children with and without ADHD may provide a developmental marker for emotional and behavioral dysregulation in adolescence, with this effect partly explained by childhood white matter organization within fronto-limbic circuitry.

## Highlights

• Childhood macrostructure in fronto-limbic networks predicts internalizing problems.
• Childhood motor ability predicts adolescent internalizing symptoms.
• This effect may be mediated by fronto-limbic macrostructure.

## Graphical abstract

Childhood motor ability predicted adolescent depressive scores in children with and without ADHD, an effect mediated by childhood macrostructure of the uncinate fasciculus. Low motor skill in children with and without ADHD may provide a developmental marker for emotional and behavioral dysregulation in adolescence, with this effect partly explained by childhood white matter organization within fronto-limbic circuitry.

**Figure:**
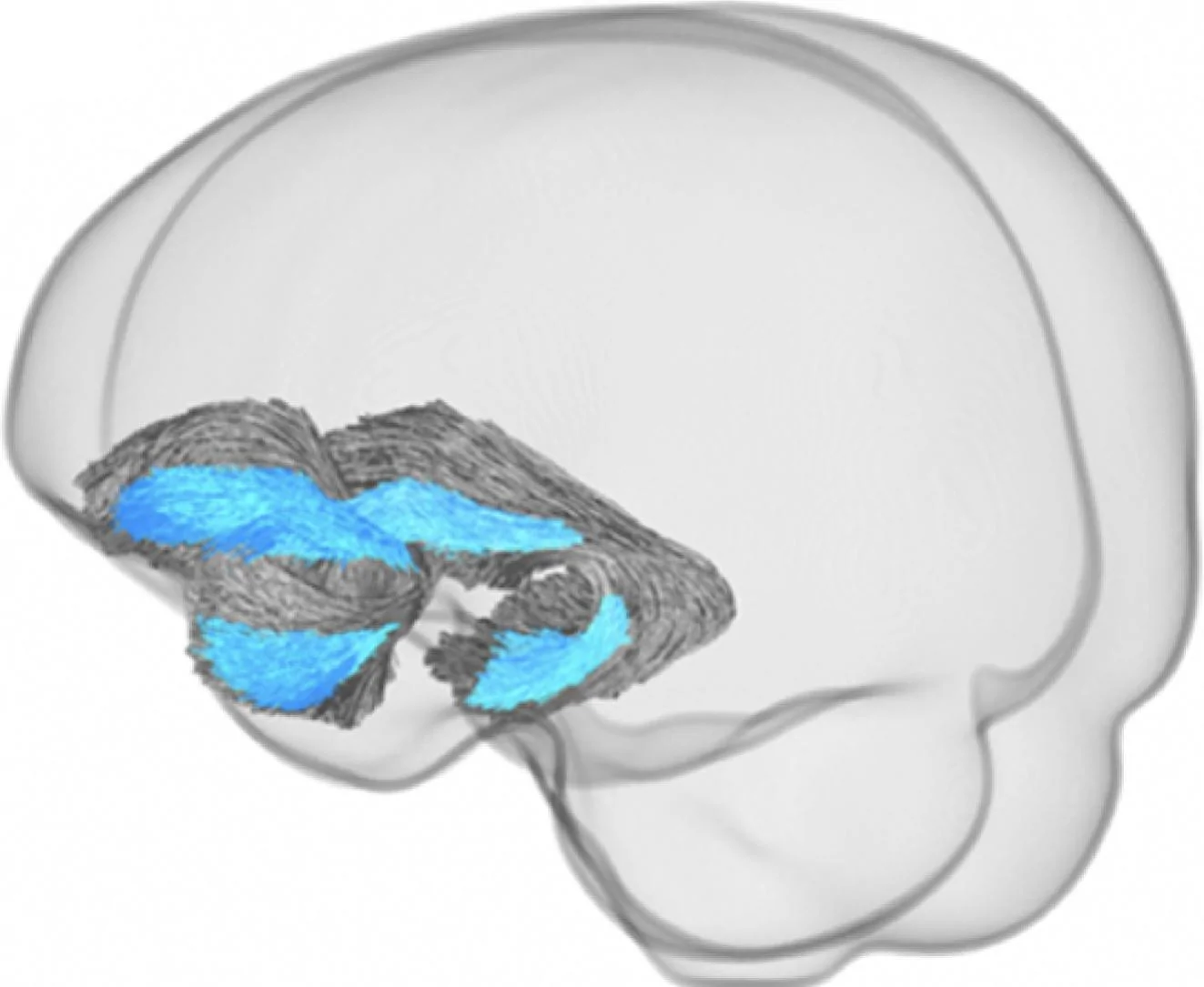

Attention deficit hyperactivity disorder (ADHD) is a highly prevalent (≈10 %) neurodevelopmental disorder, characterized by developmentally inappropriate problems with inattention and/or impulsivity/hyperactivity (Polanczyk et al., 2014). These symptoms lead to functional impairment in daily living and academic performance, and a child's capacity to develop and maintain interpersonal relationships (Polanczyk et al., 2014). Of concern, there is increasing evidence of the serious mental health consequences of ADHD, with up to 40 % of children and adolescents with ADHD experiencing significant internalizing problems (i.e. depression, anxiety, and/or social withdrawal) (see Becker and Fogleman, 2020). This is associated with decreased quality of life (Danckaerts et al., 2010), along with poorer social and academic outcomes (Blackman et al., 2005; Karustis et al., 2000). Individuals with ADHD also have poorer mortality outcomes relative to non-ADHD individuals, where deaths due to suicide, homicide and unintentional injury are significantly higher (Chen et al., 2019). Establishing objective predictors of difficulties with emotion regulation in children with ADHD at, and prior to, their emergence is critical to timely and effective intervention, and our capacity to circumvent this significant health burden.

To this end, recent longitudinal work in non-ADHD children has demonstrated that organization of white matter pathways (myelinated fibre bundles that facilitate communication throughout the brain) in the primary school-years might predict the severity of internalizing problems in adolescence (Bick et al., 2017). Similar cross-sectional effects have also been observed, with white matter morphology in pathways critical for emotional regulation (e.g. cortico-amygdala, such as the uncinate fasciculus- UF), as well as those involved in higher order cognition and sensorimotor processing (association-SLF and -ILF), predicting the severity of internalizing problems in children and adolescents (Ali et al., 2019; Andre et al., 2020; Chahal et al., 2021; Gilchrist et al., 2023). This work highlights the important role that white matter organization within the child brain may play in predicting emotional dysfunction in adolescence. In the case of ADHD, an emerging literature points towards a white matter substrate underlying symptom expression (Aoki et al., 2018; Fuelscher et al., 2021). Given this, white matter organization in children with ADHD may offer a reliable predictor for the development of internalizing problems later in life, as can be observed in non-ADHD children. However, no study has directly investigated this question in children with a diagnosis of ADHD.

Furthermore, the poorer internalizing outcomes observed in ADHD are exacerbated by the presence of co-occurring disorders of movement (Missiuna et al., 2014; Piek et al., 2007). Of concern, approximately 50 % of children with ADHD present with motor difficulties (Goulardins et al., 2017), with recent work implicating white matter organization of higher order association and projection tracts in low motor ability in this group (Hyde et al., 2023; Hyde et al., 2021). This work again provides further evidence that the involvement of white matter anomalies in ADHD may extend beyond the expression of primary symptoms (e.g. inattention and impulsivity) and contribute to secondary or co-occurring symptoms. Behaviorally, a strong body of work demonstrates a reliable association between low motor ability and internalizing problems (Mancini et al., 2019), including evidence that children with ADHD and motor difficulties are at heightened risk for developing internalizing symptoms in adolescence (Missiuna et al., 2014). This relationship is likely bi-directional. For example, motor difficulties may, in and of themselves, be developmental markers for patterns of broader brain dysfunction that directly contribute to ADHD-associated difficulties with emotional and behavioral regulation. In this case, it may be that white matter organization may mediate the longitudinal relationship between motor ability and internalizing problems in those with ADHD.

Alternatively, there is also evidence that low motor ability can precede internalizing problems in children, suggesting a potential causal effect (for a detailed discussion, see Mancini et al., 2016). To this end, the ‘environmental stress hypothesis’ (ESH) posits that low motor ability exposes children to a range of psychosocial stressors (peer conflict and victimization, poor social support, low self-esteem/efficacy, decreased physical activity etc.), which then predisposes them to developing internalizing difficulties (Mancini et al., 2019). Neurologically, longitudinal evidence shows that atypical white matter organization in infancy precedes low motor ability in childhood (Dewey et al., 2019), suggesting that white matter organization may offer a viable and valid predictor of poor motor outcomes, and that poor motor functioning then predisposes children to internalizing problems. In sum, it is similarly plausible that (i) motor ability may mediate the longitudinal relationship between white matter organization and internalizing problems in ADHD; and/or (ii) white matter organization may mediate the longitudinal relationship between motor ability and internalizing problems in those with ADHD. Given the lack of research on this topic, the present study sought to address both alternative mediating effects, to better understand the mechanisms by motor skill and white matter properties might explain the emergence of internalizing problems in adolescents with ADHD.

The initial aim of the present study was to investigate whether white matter organization in children with and without ADHD predicts the severity of internalizing problems later in adolescence. We then aimed to determine whether (i) this longitudinal effect was mediated by motor skill level, and/or (ii) the white matter organization in children with ADHD might instead mediate the expected relationship between motor skill level and internalizing problems later in adolescence. Since white matter in fronto-limbic networks (e.g. UF) and association tracts supporting higher order cognition and sensori-motor processing (e.g. ILF and SLF) have previously been implicated in childhood and adolescent internalizing problems, and have broadly been implicated childhood motor skill previously, we focused our interest on these tracts. All participants underwent diffusion MRI in childhood (aged 8–13 years), following which fixel based analyses (FBA) was performed and fibre cross-section (FC) extracted (Raffelt et al., 2017a, Raffelt et al., 2017b). Unlike those indices of white matter derived from the commonly adopted tensor model (e.g. fractional anisotropy), those derived from FBA can be attributed to a specific fibre population, improving their validity and interpretability relative to traditional tensor metrics (Dhollander et al., 2021). We then used TractSeg (Wasserthal et al., 2018) to delineate our tracts of interest, and FC was generated for fixels in each tract. Adolescent internalizing symptoms were measured between the ages of 12–18, at least 1.5 years after the childhood visit using the ‘Behavior Assessment System for Children, 2^nd^ Edition’ (BASC- Reynolds, 2010). Since adolescent reports of internalizing problems may differ between the individual and their care-giver (Youngstrom et al., 2000), we included both ‘parent’ and ‘child’ report measure of the BASC.

To address our first aim, we investigated whether childhood white matter organization predicts adolescent internalizing symptoms in youth with and without ADHD diagnosed in childhood. We hypothesized that greater FC in the uncinate fasciculus and the ILF and SLF in childhood would predict greater adolescent internalizing problems. Finally, where these hypotheses were supported, we hypothesized that (i) motor skill level would mediate the relationship between childhood white matter organization and adolescent internalizing problems. Similarly, given the bi-directional nature of the relationship between white matter organization and motor skill, we also expected that childhood white matter organization would mediate the expected relationship between early motor ability and adolescent internalizing problems.

## 1 Methods

### 1.1 Participants

The present sample included 40 children with ADHD aged between 8.02 and 12.8 years (*M*_Age_: 10.1, *SD*_Age_: 1.40; Female = 11), and 36 typically developing (TD) controls aged between 8.42 and 13.0 years (*M*_Age_: 10.3, *SD*_Age_: 1.16; Female = 14). Participants had an initial visit (Time 1) in childhood (aged 8.02–13.0 years) where MRI took place (see below), followed by a follow-up visit (Time 2) during adolescence (aged 12.1–18.0 years) at least 1.5 years after the initial childhood visit (range = 1.57 to 8.87 years, *M*_diff_ = 4.45 years, *SD*_diff_ = 1.93 years) (see Table 1 for a summary of demographic and clinical features). We note that the present sample was taken from a larger cohort study, whereby participants may have been involved in several follow-up visits. Here, ‘Time 2’ reflects the assessment follow-up at the oldest age.

**Table 1:** Demographic and clinical characteristics of study participants. Values represent means and (standard deviations) unless otherwise noted.

	ADHD						non-ADHD						ADHD vs. non-ADHD group comparison	
	Females	*n*	Males	*n*	Total	*n*	Females	*n*	Males	*n*	Total	*n*	*Statistic*	*p*
Demographics														
Age (T1)	9.40 (1.27)	11	10.37 (1.37)	29	10.10 (1.40)	40	10.13 (1.08)	14	10.46 (1.22)	22	10.30 (1.16)	36	*U* = 632.5	0.440
Age (T2)	14.96 (1.73)	11	14.60 (1.44)	29	14.70 (1.51)	40	14.49 (2.01)	14	14.71 (2.02)	22	14.60 (1.99)	36	*U* = 702.0	0.856
Race A:B:M:W (%)	0:36:9:54	11	0:18:14:68	28	0:23:13:64	39	7:7:29:57	14	14:18:14:54	22	11:14:19:56	36	χ^2^ = 5.92	0.116
SES	51.1 (10.6)	11	50.4 (14.2)	29	50.6 (13.2)	40	53.3 (9.3)	14	51.2 (14.9)	22	52.0 (12.9)	36	*U* = 686.0	0.727
WISC IQ	114.6 (15.7)	11	118.4 (12.0)	29	116.5 (13.1)	40	114.5 (11.5)	14	114.9 (13.3)	22	114.7 (12.5)	36	*U* = 627.0	0.335
Conners DSM Hyp/Imp	74.3 (17.7)	11	70.3 (9.5)	29	71.4 (12.2)	40	45.9 (6.7)	14	43.5 (5.4)	22	44.5 (6.0)	36	*U* = 45.0	<0.001
Conners DSM Inattention	71.4 (16.4)	11	67.0 (12.1)	29	68.2 (13.4)	40	46.4 (5.5)	14	46.6 (5.8)	22	46.5 (5.6)	36	*U* = 92.5	<0.001
Stimulant medication (n,%)	5 (45.5)	11	19 (65.5)	29	24 (60.0)	40	–		–		–		–	–
Comorbidities														
ODD (n)	8	–	7	–	15	–	0	–	0	–	0	–	–	–
Anxiety (n)	3	–	4	–	7	–	1	–	0	–	1	–	–	–
Depression (n)	0	–	1	–	1	–	2	–	0	–	2	–	–	–
Phobia (n)	6	–	3	–	9	–	1	–	0	–	1	–	–	–
OCD (n)	1	–	0	–	1	–	0	–	0	–	0	–	–	–
Adjustment Disorder (n)	0	–	0	–	0	–	0	–	1	–	1	–	–	–
Panic Disorder (n)	0	–	1	–	1	–	0	–	0	–	0	–	–	–

Notes: Mean (SD) reported unless otherwise noted. Conners DSM Hyp/Imp = T-score for the DSM hyperactivity/impulsivity from the Conners-R (*n* = 36) or Conners-3 (*n* = 39) Parent Report scale WISC IQ: General Ability Index for the Wechsler Intelligence Scale for Children 4th (*n* = 68) or 5th (*n* = 18) Edition. NOTE: Race: A: B: M: W (Asian: Black: Multiracial; White); SES- Socio-economic status; ODD- Oppositional defiant disorder; OCD- Obsessive compulsive disorder.

**Fig. 1:**
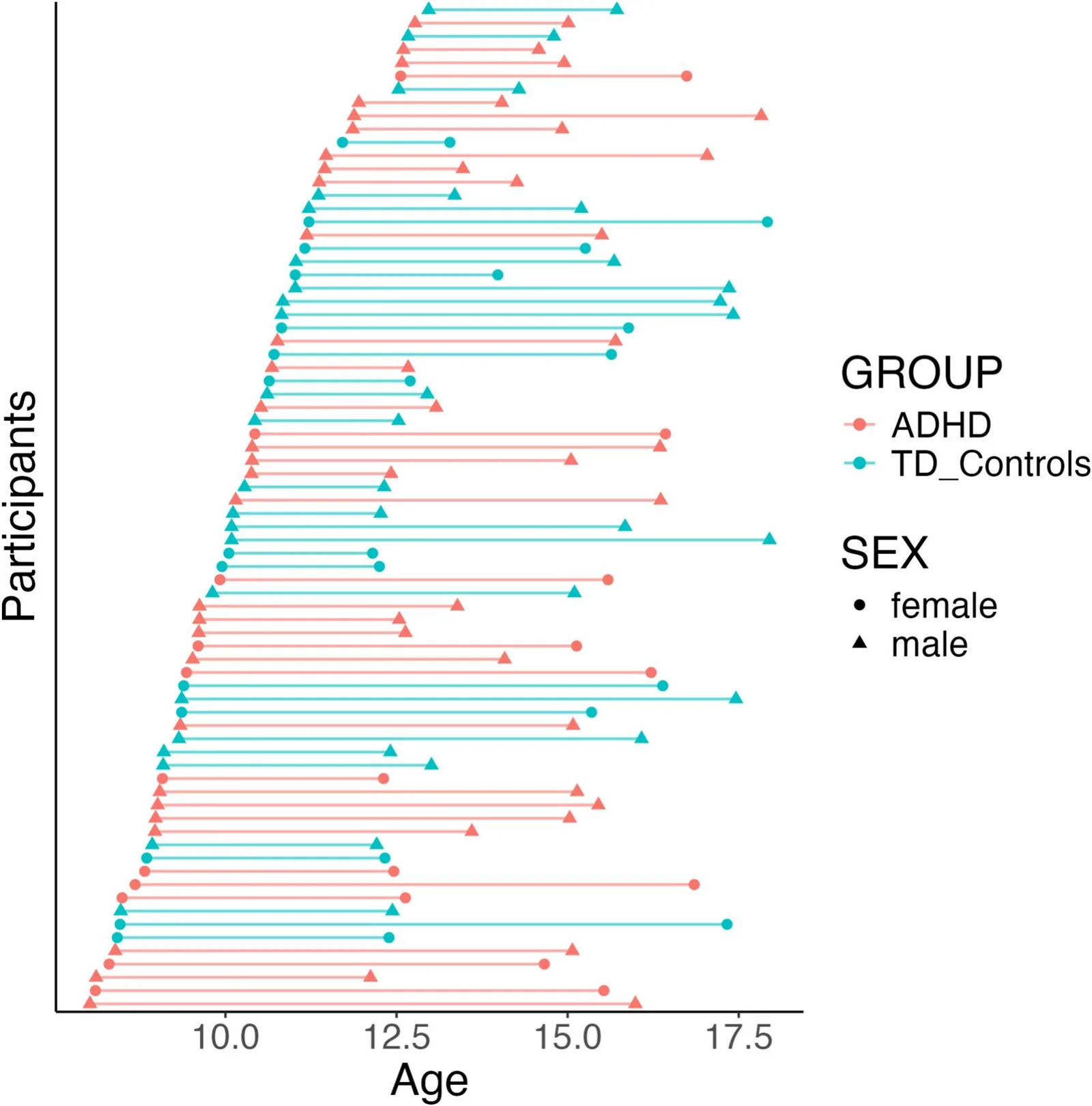
Age and sex distribution for adolescents with ADHD (n = 40, no. of observations = 80) and TD controls (n = 36, no. of observations = 72). Each dot represents a timepoint.

Fig. 1 illustrates the spread of observations for the ADHD and TD control groups across the two timepoints. Participants were recruited through local schools and community centres using a combination of onsite advertisements, school mailings, electronic medical records, and word of mouth. Children with ADHD met full DSM-IV or DSM-5 criteria according to the following: Firstly, they were given a diagnosis of ADHD on the Kiddie Schedule for Affective Disorders and Schizophrenia (K-SADS: Kaufman et al., 1997); this included detection of at least 6/9 symptoms of inattention, hyperactivity or both and cross-situational impairment. Secondly, children obtained a T score of 65 or above on the Conners-R (*n* = 36) or Conners-3 (*n* = 39) DSM Inattention or Hyperactivity/Impulsivity Scales (Conners, 1997).

The clinical features of participants were characterized using the Diagnostic Interview for Children and Adolescents (*n* = 53) (DICA-IV: Reich, 2000) or K-SADS) (*n* = 22) at baseline, whereas only the K-SADS was administered for the adolescent follow-up. Diagnostic interviewing was conducted by a Master's level clinician, with information integrated from the above rating scales in order to confirm a diagnosis of ADHD. This occurred under the supervision of licensed clinical psychologists (KSR, KES) or a neurologist (SHM) with extensive experience diagnosing ADHD in children and adolescents. Children in the non-ADHD group did not present with a history of either a neurodevelopmental or mental health disorder (other than specific phobia), nor did they meet criteria for these disorders on the DICA-IV or K-SADS. Further, they scored below the clinical cut-offs for ADHD (*T* < 60) on the above Conners rating scales.

General exclusionary criteria (regardless of group membership) were a history of seizures, head injury, or neurodevelopmental disorder other than ADHD (e.g., autism spectrum disorder, language-based learning disorders), and a general ability index (GAI; a composite index score of intellectual reasoning ability without working memory and processing speed index scores included, as these are often affected in individuals with ADHD and impact the full-scale intelligence quotient score) below 80. The present project received ethical approval from the Johns Hopkins Institutional Review Board, and all parents gave informed consent to their children's participation, while children gave assent.

### 1.2 Internalizing problems

**Fig. 2:**
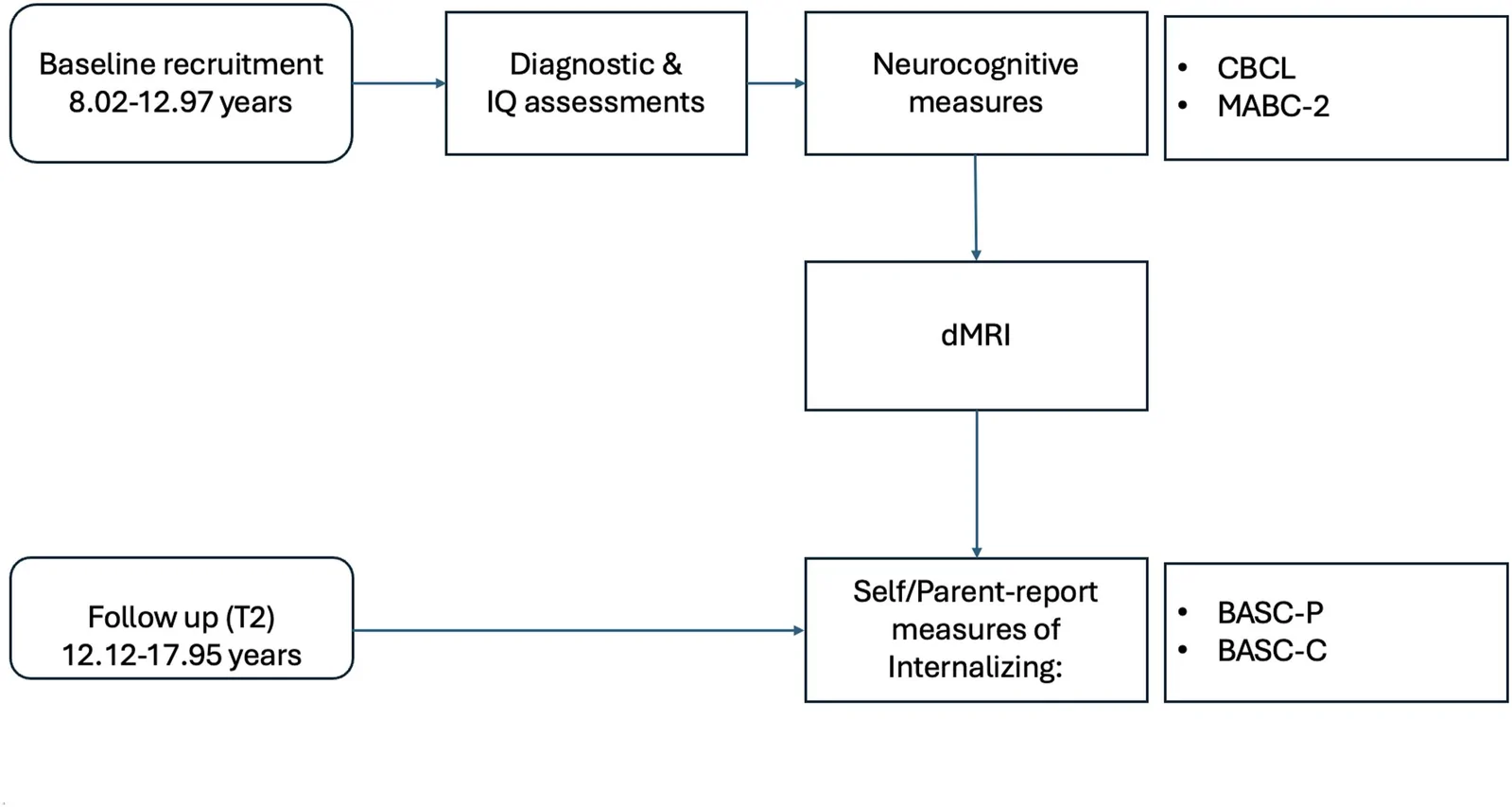
Flowchart of the assessment procedure for the current study. Note: ADHD: Attention-Deficit/Hyperactivity Disorder; CBCL: Child Behavior Checklist; MABC; Movement Assessment Battery for Children; dMRI: Diffusion MRI; BASC-P: Behavior Assessment System for Children (parent report); BASC-C: Behavior Assessment System for Children (child report).

At Time 2, The Behavior Assessment System for Children (BASC: Reynolds, 2010) child and parent report versions were administered to measure the presence of internalizing problems. The ‘depression’, ‘anxiety’, and ‘somatization’ subscales were the focus of the present study, with raw scores converted to age- and sex-adjusted *T* scores. At Time 1, internalizing problems were measured using the Child Behavior Checklist (CBCL: Achenbach, 1999), parent report. Again, the ‘affective’ (i.e. dysthymic and depressive symptoms), ‘anxiety’, and ‘somatization’ subscales were of interest to the present study, with raw scores converted to age- and sex-adjusted *T* scores (See Fig. 2).

### 1.3 Motor skill

Motor ability was assessed at Time 1 using the Movement Assessment Battery for Children-2 (MABC-2: Henderson et al., 2007), a well-validated standardized measure of motor ability. Participants complete tasks of manual dexterity (3 items), aiming and catching (2 items) and balance (3 items), with their performance converted to a percentile relative to a normative sample.

### 1.4 Neuroimaging

At Time 1, all participants underwent diffusion MRI, initially completing a mock MRI session to acclimate them to the MRI environment to reduce anxiety. MR scanning was conducted on a Philips 3 T scanner (Philips Medical Systems, Best, The Netherlands) using either an 8- or 32-channel head coil. In order to determine cerebral volume for subsequent analyses (see below), high resolution T1-weighted, MPRAGE images were acquired in the axial plane (slice thickness 1.00 mm; FOV 26 cm, acquisition matrix 256 × 256). Diffusion weighted images (DWI) were acquired using a single-shot echo-planar sequence with sensitivity encoding (TR: 6.356 s, TE: 75 ms, sensitivity encoding acceleration factor 2.5, 2.2 mm axial slices, acquisition matrix 96 × 96 [FOV 212 mm]). Two diffusion sequences were collected for each participant, with 32 non-collinear diffusion encoding directions (b value 700 s/mm^2^ and one b0 for each run).

Though it is preferable to conduct FBA using high angular resolution diffusion imaging (HARDI) (i.e. b values of >3000, > 45 directions), recent consensus work demonstrates the feasibility of conducting CSD based FBA on diffusion sequences with more modest *b* values (such as those of the current study) (Dhollander et al., 2021). Indeed, FBA remains preferable to tensor-based analyses in these scenarios, the former of which are unable to reconcile multiple fibre populations within a given voxel. Conversely, FBA offers reliable fixel specific analysis of effects of interest (see Dhollander et al., 2021 for a detailed discussion).

Using the T1 acquired image, total cerebral volume (TCV) was generated for each participant, and adopted as a covariate as necessary. Specifically, we adopted FreeSurfer's ‘recon-all’ command (Fischl et al., 2004), and TCV was generated for each participant. A Mann-Whitney *U* test revealed no significant group differences in TCV across adolescents with and without childhood ADHD (*U* = 685.00, *p* = 0.72, *r* = 0.05). Further, following comparable work adopting FBA in children and adolescents (e.g., Genc et al., 2020), individual differences in head motion were estimated in each participant's diffusion sequence (excluding b0 volumes) using mean framewise displacement (FWD) in FSL (Power et al., 2012). We failed to detect any correlation between FC in our tracts of interest and FWD. Similarly, no group differences in FWD were detected (see Section A of the Supplementary materials for relevant analyses). Given this, we did not correct for FWD in subsequent analyses.

*As per our earlier work* (Hyde et al., 2024), diffusion data were pre-processed using the FSL Diffusion Toolbox. FSL's TOPUP tool was used to conduct susceptibility distortion corrected (Andersson et al., 2003; Smith et al., 2004), using the first b0 image and a structural T2-weighted image with the same geometry as the diffusion data but different phase-encoding direction (right-left). Following this, eddy current-induced distortions, between-volume head motion, and outlier detection and replacement was conducted using FSL's EDDY utility (Andersson et al., 2016; Andersson and Sotiropoulos, 2016). Following pre-processing, data were analyzed using MRTrix3 (Tournier et al., 2019) and MRTrix3Tissue (https://3Tissue.github.io), a fork of MRTrix3. As per the recommended FBA pipeline, data were bias field corrected and upsampled to an isotropic voxel size of 1.30 mm^3^. Group average response functions for gray matter, white matter and CSF were then used to generate individual FOD maps using single-shell 3-tissue constrained spherical deconvolution (SS3T-CSD) (Dhollander et al., 2016). Intensity normalization then took place on FOD images to ensure consistency of FOD magnitudes across participants (Raffelt et al., 2017a, Raffelt et al., 2017b).

A study specific population FOD template was generated based on 20 representative participants from the ADHD Group (M_age_ = 10.44, 10 females) and 20 representative participants from the non-ADHD group (M_age_ = 10.12, 10 females). These participants were selected from the larger cohort of 339 MRI scans. To facilitate comparison with previous work this template was subsequently registered to MNI space. All 76 participant FOD images were registered to the study specific FOD template. Fibre-cross section (FC _log_) was then calculated for each participant- FC provides a macroscopic measure of the cross-section perpendicular to the fibre (Raffelt et al., 2017a, Raffelt et al., 2017b). FC _log_ was generated to ensure normality since FC is commonly skewed.

It is also possible to generate a fibre density (FD) measure following FBA, the former being a microscopic measure of the intra-axonal volume of a fibre population. Though it is feasible to calculate FD based on the diffusion parameters of the current study, signals from the extra-cellular space beyond the axon can contribute to the FD metric at lower b values (1000 s/ms^2^ or similar), which may bias it (see Dhollander et al., 2021). This can hinder interpretation. As such, FD was not calculated in the present data set, nor was fibre-density- cross section (FDC), the product of FC and FD.

Tracts of interest to the present study were the SLF (bilateral), ILF (bilateral) and uncinate fasciculus (bilateral). To delineate these tracts, we used the automated TractSeg method (Wasserthal et al., 2018) in the population FOD template to segment those voxels that corresponded to SLF (combined from the SLF I, II and III projections in the TractSeg library), ILF and the UF within each hemisphere.

### 1.5 Design and analysis

**Predicting adolescent internalizing problems from childhood white matter organization.** The initial aim of our study was to determine whether childhood white matter organization could predict adolescent internalizing problems in those with and without ADHD. Since the latter was assessed using the BASC, which contained both parent and child reports, we opted to first investigate whether Time 1 white matter organization could predict Time 2 internalizing problems, as measured by the *parent* report BASC ‘anxiety’, ‘depression’, and ‘somatization’ subscales. We then replicated these analyses using the Time 2 *child* report BASC ‘anxiety’, ‘depression’, and ‘somatization’ subscales. Given that parent and child reports for psychological well-being often differ (Youngstrom et al., 2000), this allowed us to determine whether the relationship between childhood (Time 1) white matter organization and adolescent (Time 2) internalizing problems differed depending on whether the latter were measured from the perspective of the parent or child.

In preparation for the relevant statistical analyses, participants' FC maps were cropped to correspond to fixels belonging to our tracts of interest. This resulted in 6 fixel maps corresponding to our tracts of interest (left and right SLF, ILF and UF). These fixel maps were included in our subsequent analyses. To investigate the relationship between white matter organization in our tracts of interest (Time 1) and internalizing problems at Time 2, we used the connectivity-based fixel enhancement (CFE) method to investigate the relationship between FC _log_ in tracts of interest and ‘anxiety’, ‘depression’, and ‘somatization’ BASC scales (parent report) in separate analyses (Raffelt et al., 2015). This was conducted with all participants included (i.e., combined ADHD and TD control). In these analyses, we covaried for sex, age, and TCV. The CFE is the optimum approach for investigating correlations between white matter and behavioral outcome, with *p* values provided for each fixel which are permutation-based and family-wise error corrected (Genc et al., 2020; Raffelt et al., 2015). Within tracts, statistical significance for correlations was set at *p* < 0.05 family wise error (_FWE_) corrected. For each tract and hemisphere, we further inspected the FWE corrected *p*-value maps to ensure that significant fixels did not appear in isolation (these effects were considered spurious).

As noted above, we did not observe an association between FC and FWD, nor were there group differences in FWD. As such, FWD was not included as a covariate in the above CFE analyses, Still, in order to confirm the robustness of our findings, where the CFE analysis showed a significant association between FC in a given tract and parent or child report internalizing problems, we replicated the CFE analyses controlling for FWD. In each case, there was no appreciable difference in the region of effects reported where we controlled for FWD relative to the effects observed here in the manuscript where we did not. Indeed, there was almost entire overlap in the regions implicated. Further, the correlation between the mean FC in those regions where a significant association was observed between FC and an internalizing symptom when FWD was and was not controlled for ranged between (*r* = 0.87–0.99) (see Section A of the Supplementary materials for relevant analyses), suggesting almost total overlap in the effects observed when controlling for FWD relative to when not doing so. As such, we can be confident that the effects reported here are robust to the effects of head motion.

Given our sample size, our design was not optimized for parametrically testing whether any observed associations between FC in our tracts of interest and BASC (parent) scores differed according to group membership (ADHD vs TD control). Still, we visualize this effect for transparency and present the association between mean FC (averaged across the significant fixels from the CFE analysis) and BASC (parent) scores separately in the ADHD and TD control groups in our results and supplementary sections as appropriate. While these visualizations are presented below, for transparency we have included these figures with the accompanying R and *p* values for each group in Section B of the Supplementary materials. However, for the reasons stated above, we urge caution in their interpretation given the modest sample size in each group (particularly *p* values). Further, while not a primary aim of the study, we also thought it relevant to investigate whether any observed effect between childhood (Time 1) white matter organization and adolescent (Time 2) internalizing problems held after controlling for initial (Time 1) levels of childhood internalizing symptoms, to account for baseline differences in internalizing symptoms. We present these analyses in the Section C of the Supplemental materials. Note that since internalizing problems at Time 1 were measured using the CBCL (rather than the BASC), the CBCL subscales (i.e., anxiety, affective [viz depression/dysthymia], and somatization) were used to control for Time 1 anxiety, depression and somatization respectively.

**Comparing white matter organization in childhood among ADHD and TD control groups.** To better understand these predictive relationships, we tested whether there were group differences in white matter organization between those with and without ADHD in white matter regions where we had observed an association between childhood white matter organization (Time 1) and BASC internalizing scores (parent report) at Time 2. This was achieved by using linear mixed-effects (LME) modelling with restricted maximum likelihood estimation (REML). Here, the dependent variable was mean FC (as described above), and tract and group were entered as fixed effects. Further, age, sex and TCV were entered as covariates. Finally, all models included a random intercept to control for clustering of FC values within each participant. All of the above analyses were replicated with the relevant ‘Child Report’ Time 2 BASC Internalizing scores replacing ‘Parent Report’.

**Mediation analysis.** Finally, in those cases where white matter organization in childhood (Time 1) was found to be significantly associated with adolescent (Time 2) internalizing problems, we tested whether this effect was mediated by motor skill. As is recommended of mediation analyses, we first ensured that our predictor (e.g. *X*- white matter organization) and outcome measures (e.g. *Y*- internalizing problems) were correlated, that our predictor and the mediator [e.g. *M*-motor skill (i.e. MABC-2 percentile rank)] were correlated, and that our mediator and outcome were correlated. These correlation matrices can be found in Section F of the Supplemental materials. Where the above assumptions were met, mediation regression was conducted, with entered variables residualized for age, sex and TCV. Where these assumptions were not met, mediation analyses were not conducted.

**Behavioral Comparisons.** Given earlier accounts that have suggested greater internalizing problems in ADHD, we initially compared the severity of internalizing problems between those with and without ADHD at Time 2. Here, we compared the mean depression, anxiety and somatization scores from the BASC between groups. This was conducted separately for both the ‘parent’ and ‘child/self-report’ report versions of the BASC. Though not a primary aim of our study, to facilitate comparisons of the present work with earlier accounts, we compared motor ability between groups at Time 1. Specifically, we compared mean percentile ranking on the MABC-2 for ADHD and non-ADHD groups.

While not a direct focus of the present study, prior work from our group has identified sex differences in neural and behavioral outcomes in males and females with ADHD (DeRonda et al., 2021; Rosch and Mostofsky, 2016; Rosch et al., 2018; Tang et al., 2019). The sample size of the present study precluded us from investigating the degree to which sex differences might be driving the biological associations between childhood white matter and adolescent internalizing problems described next. Still, in order to facilitate comparison with earlier work, where significant group differences were identified in the above behavioral measures, we further examined possible sex differences in within our ADHD and TD control group respectively in an exploratory analysis which can be found in Section E of the Supplemental materials. The assumption of normality (according to Shapiro Wilks), and the assumption of homogeneity of variance (according to Levene's Test) was violated in most cases for the above comparisons. Accordingly, a series of Mann-Whitney *U* tests were conducted for group comparisons.

Analyses were conducted in Jamovi version 2.3.28, while scatterplot images were generated using R version 4.4.1 (for relevant R code, see https://github.com/chydeDeakin/Hyde_et_al_2026.git. As above, FBA were conducted according to the recommended MRTrix pipeline. Finally, summary statistics for demographics, as well as LME models and mediation analyses were conducted in Jamovi).

## 2 Results

### 2.1 Predicting adolescent internalizing problems from childhood white matter organization

**Fig. 3:**
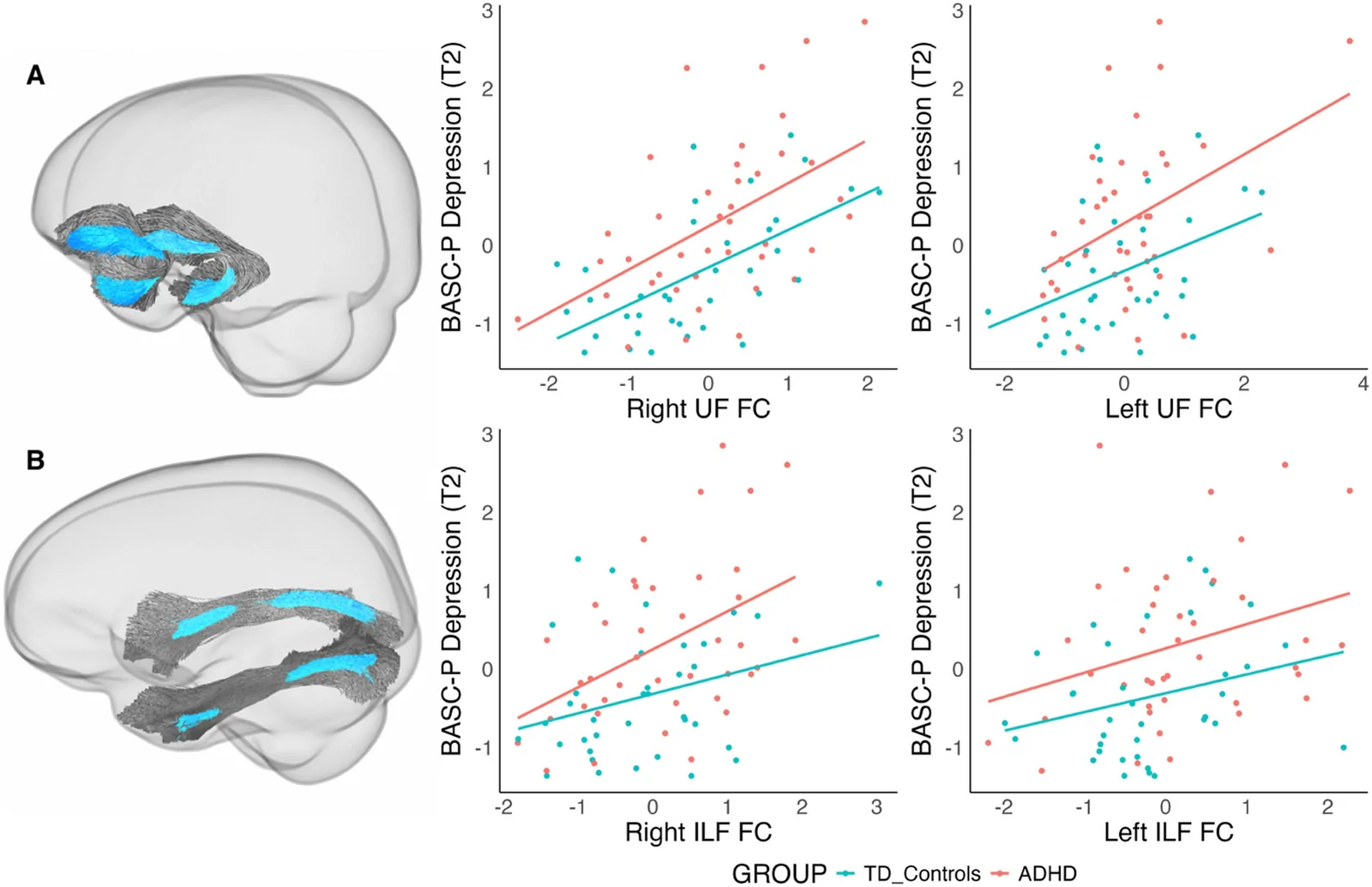
Tract specific streamlines showing a significant positive association between FC of the (A) bilateral UF and (B) bilateral ILF during childhood with scores on the parent report BASC-P Depression subscale in adolescence. Scatterplots show the linear relationship between FC of the bilateral UF and ILF for each group.

**Parent report data.** Tract of interest FBA revealed a significant positive association between FC of the bilateral UF and ILF in childhood with BASC-P depression scores in adolescence. Fig. 3 shows streamline segments associated with fixels demonstrating a significant increase in FC (*p* < 0.05 _FWE_) in the bilateral (A) UF and (B) ILF respectively. Scatterplots of mean FC for each participant (averaged across significant fixels from the CFE analysis) and BASC-P depression scores illustrate that ADHD-diagnosed adolescents demonstrated higher FC than the non-ADHD group. No significant associations were observed for FC with respect to scores on the anxiety and somatization subscales of the BASC-P. When covarying for Time 1 (childhood) CBCL depression scores, only the positive association between FC of the bilateral UF and depression remained significant (*p* < 0.05 _FWE_, Supplemental materials, Section C). We did not find any significant associations between adolescent internalizing scores with white matter properties underlying the bilateral SLF during childhood.

**Fig. 4:**
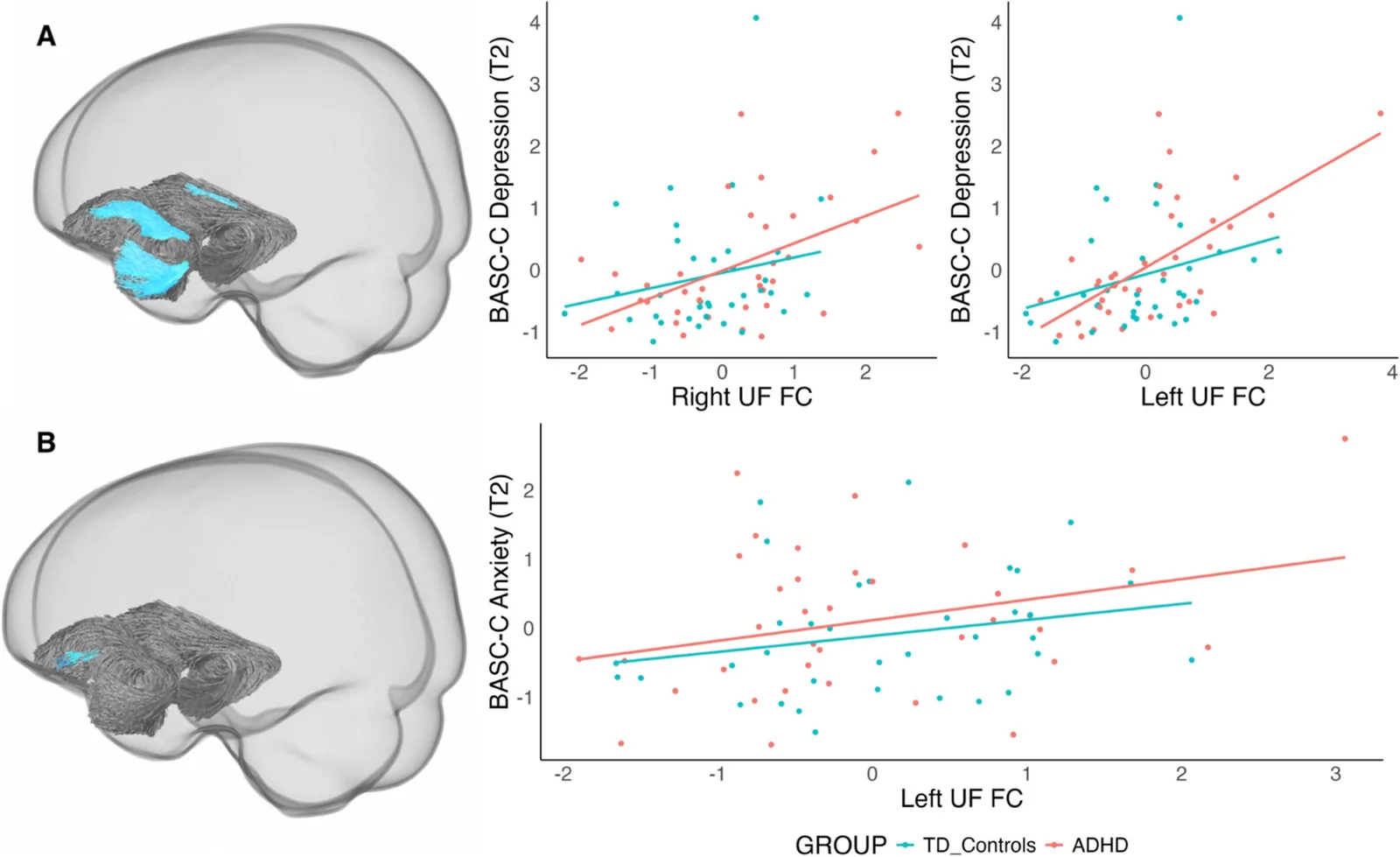
Tract specific streamlines showing a significant positive association between FC of the (A) bilateral UF during childhood with scores on the self-report BASC-C Depression subscale during adolescence and (B) left UF during childhood with scores on the self report BASC-C Anxiety subscale during adolescence. Scatterplots show the linear relationship between FC of and Internalizing scores for each group.

**Child (self-report) data.** Results for the self report BASC-C show a significant positive association between FC of the bilateral UF and depression scores (*p* < 0.05 _FWE_, see Fig. 4, panel A). Furthermore, we also observed a significant positive association between anxiety scores and FC of the left UF (*p* < 0.05 _FWE_, Fig. 4, panel B). The positive relationship between childhood left UF FC and adolescent depression scores remained significant after covarying for CBCL depression scores at childhood Time 1 (*p* < 0.05 _FWE_, Supplemental materials, Section C). As with the parent report analysis, no significant associations were found for white matter underlying the bilateral SLF and internalizing scores on all subscales.

### 2.2 Group differences in fibre cross-section between adolescents with and without ADHD

**Fig. 5:**
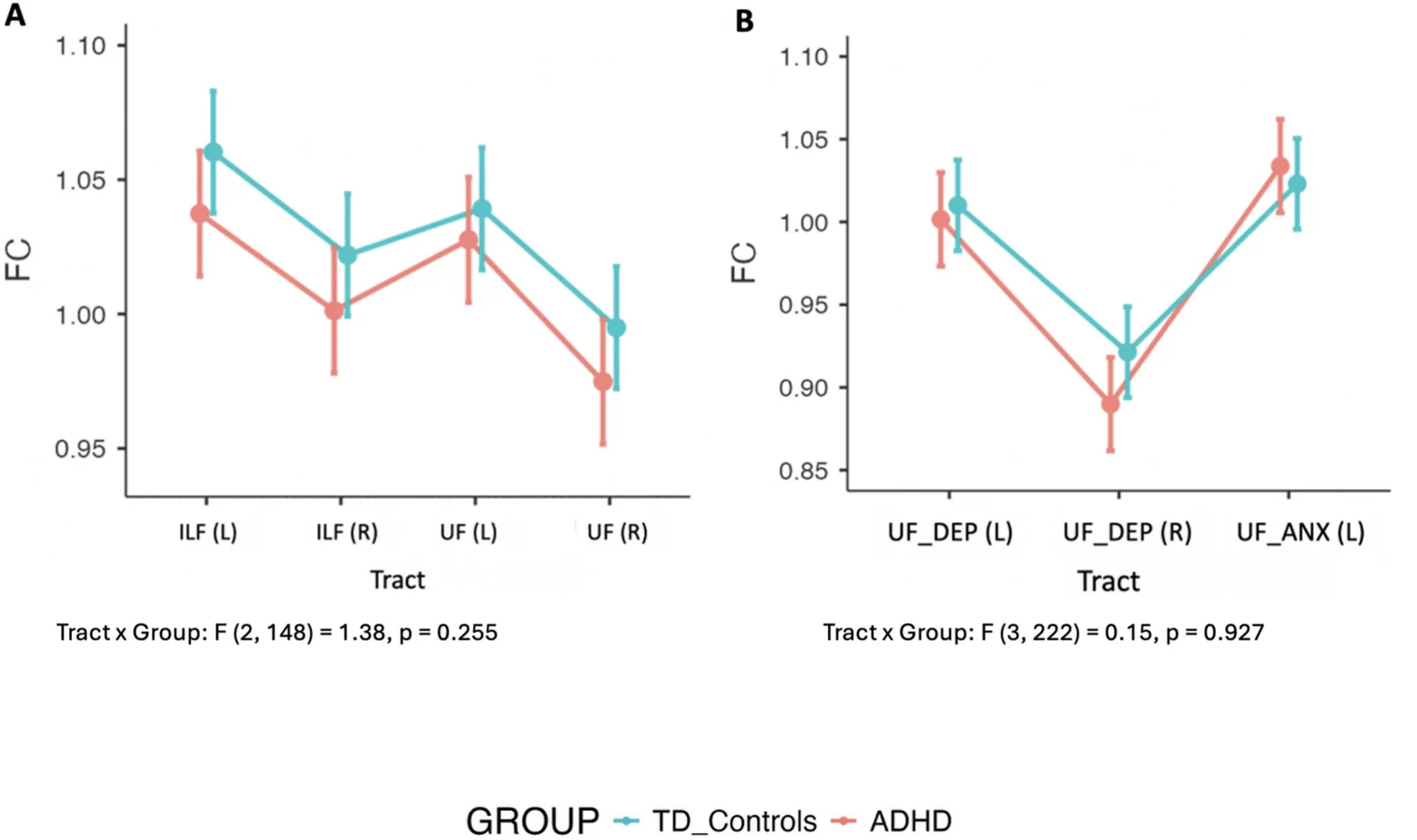
Effect plots demonstrating group differences in FC in those regions where CFE revealed an association between FC during childhood and internalizing problems during adolescence as measured by (A) parent (BASC-P) and (B) self (BASC-C) report scores. For linear mixed-models involving the BASC-C, we applied a combined model involving significant fixels from the BASC-C Depression (UF_DEP) and Anxiety (UF_ANX) CFE analyses.

For models involving significant fixels from the BASC-P (ie depression scale) CFE analysis, the full model was significantly better than the one with only the intercept and covariates, χ2 (7) = 55.74, *p* < 0.001. There was a significant main effect of tract (bilateral ILF and UF), TCV and age on FC. In contrast, there was no significant main effect of sex. A trend towards significance was identified for the main effect of group (*p* = 0.12), such that FC tended to be lower in the ADHD group (Fig. 5A). However, this effect did not reach statistical significance. There was no evidence of an interaction effect between tract and group.

Additionally, for models involving significant fixels from the BASC-C (ie depression and anxiety scales) CFE analysis, the full model was also significantly better than the one with only the intercept and covariates, χ2 (5) = 83.93, *p* < 0.001. There was a significant main effect of tract (UF left and right (depression), UF right (anxiety)) and TCV on FC, whilst no significant main effect of sex or age was identified (Fig. 5B). Lastly, the main effect for group and the interaction between tract and group were not significant. Fig. 5 plots the pattern of tract by group effects in FC between ADHD and non-ADHD cohorts for CFE analysis involving (A) BASC-P and (B) BASC-C internalizing scores. Omnibus tests for the full models are presented in Section D the Supplemental materials.

### 2.3 Behavioral comparisons

Group comparisons on behavioral measures are presented in Table 2. Results of the Mann-Whitney *U* tests for group difference indicated that the adolescents with ADHD group showed significantly higher depression scores in adolescence (Time 2) on the parent report BASC-P (but not child report) compared to adolescents without ADHD. No significant group differences emerged for somatization or anxiety. Further, there were no significant group differences between adolescents with and without ADHD on the BASC-C for somatization, depression, or anxiety. Lastly, TD children reported significantly higher motor competency (MABC-2) scores compared to ADHD children. Results of sex differences in behavioral measures are presented in Section E of the Supplemental materials.

**Table 2:** Group means, standard deviations and Mann Whitney *U* difference tests for all behavioral variables of interest.

	ADHD			non-ADHD					
	*n*	*M*	*SD*	*n*	*M*	*SD*	*U*	*p*	r
BASC-P Depression	40	53.63	10.61	36	47.11	7.14	459.50	<0.01	0.36
BASC-P Somatization	40	51.28	12.84	36	47.31	8.37	625.50	0.33	0.13
BASC-P Anxiety	40	49.50	10.41	36	47.47	10.98	626.00	0.33	0.13
BASC-C Depression	36	45.81	6.01	35	44.40	6.26	519.50	0.20	0.18
BASC-C Somatization	36	48.92	10.04	35	46.60	7.25	578.00	0.55	0.08
BASC-C Anxiety	36	48.67	11.07	35	46.34	10.23	547.50	0.35	0.13
MABC-2	36	−0.33	0.95	36	0.33	0.91	385.00	<0.01	0.41

Notes: BASC-P: The Behavior Assessment System for Children (parent report); BASC-C: The Behavior Assessment System for Children (child/self report); MABC-2: Movement Assessment Battery for Children-2.

### 2.4 Mediation analysis

A series of mediation analyses were planned to examine the explanatory effect of motor competency on the relationship between white matter organization during childhood and internalizing problems during adolescence. For analyses involving the parent report BASC-P, assumption testing demonstrated significant partial correlations (controlling for age, sex and TCV) between the following: (1) FC of the right ILF during childhood with childhood MABC-2 scores and adolescent BASC-P Depression scores; and (2) FC of the right UF during childhood with childhood MABC-2 scores and adolescent BASC-P Depression scores (for partial correlation matrices, see Section F of Supplementary materials- S4). Thus, mediation analyses were conducted on these relationships (presented next).

Neither MABC-2 scores nor FC were significantly associated with scores on the depression and anxiety subscales of the BASC-C. Therefore, we did not analyse the mediating effect of motor competency on the relationship between childhood FC and adolescent internalizing problems (self-report) (for partial correlation matrices, see Section F of Supplementary materials- S5).

### 2.5 Effect of motor competency on the relationship between right ILF FC and parent report depression

**Fig. 6:**
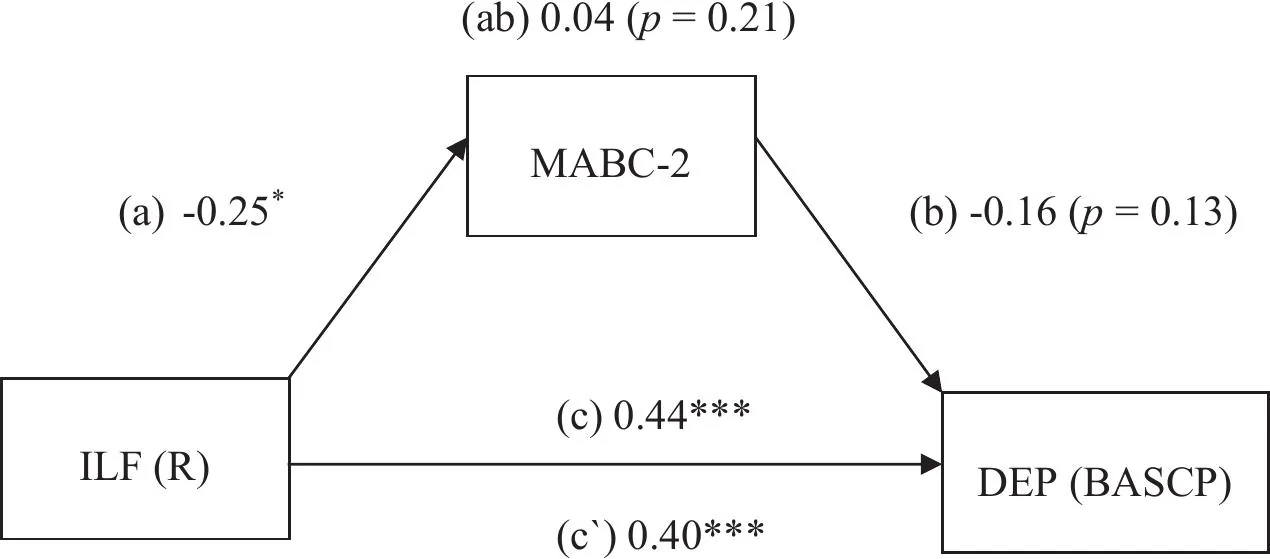
Mediation model depicting mediation of childhood MABC-2 on the relationship between right ILF FC during childhood and BASC-P Depression scores during adolescence. Note: (a): IV-mediator; (b) mediator-DV; (c’) direct effect of IV-DV; (c) total effect of IV-mediator-DV; (ab) indirect effect of mediator on the IV-DV; DEP (BASC-P): The Behavior Assessment System for Children (parent report) Depression subscale; MABC-2: Movement Assessment Battery for Children-2; ILF (R): Right Inferior Fasciculus Fibre cross-section; All values are beta coefficients. Note. * *p* < 0.05 ** *p* < 0.01, *** *p* < 0.001.

Results of the mediation analysis looking at the effect of childhood MABC-2 scores on the relationship between FC of the right ILF during childhood with BASC-P depression scores during adolescence are presented in Fig. 6. As Fig. 6 illustrates, results demonstrated a non-significant indirect effect (*β* = 0.04, *p* = 0.21), suggesting that MABC-2 scores did not mediate the significant association between white matter during childhood and depression scores on the BASC-P during adolescence. Further model details can be accessed in Section F of the Supplementary Materials (S6).

### 2.6 Effect of motor competency on the relationship between right UF FC and parent report depression

**Fig. 7:**
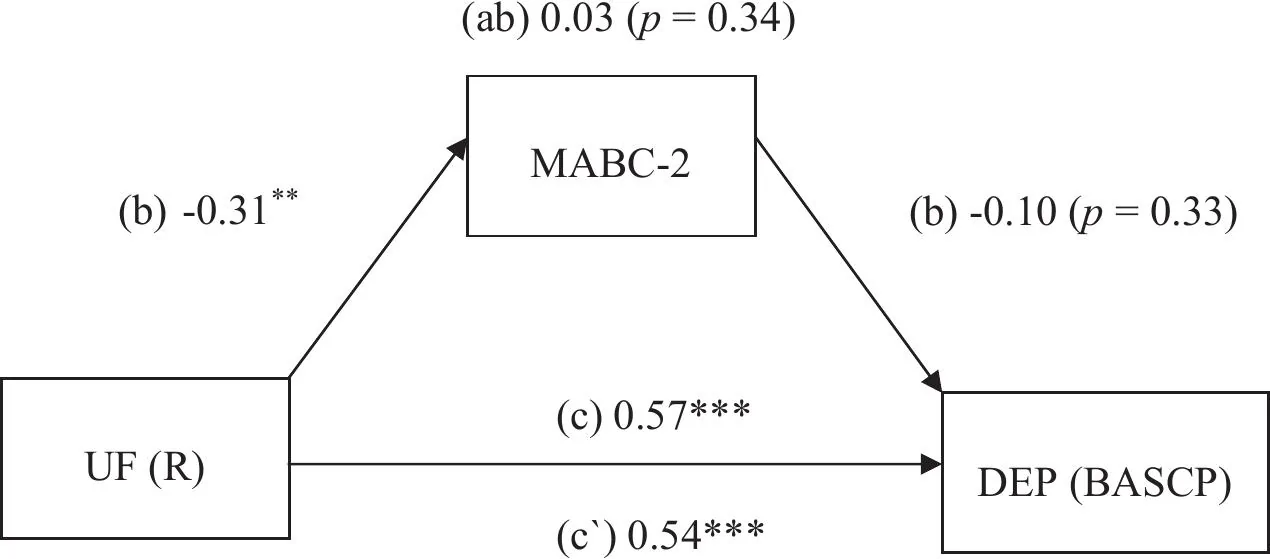
Mediation model depicting mediation of childhood MABC-2 on the relationship between right UF FC during childhood and BASC-P Depression scores during adolescence. Note: (a): IV-mediator; (b) mediator-DV; (c’) direct effect of IV-DV; (c) total effect of IV-mediator-DV; (ab) indirect effect of mediator on the IV-DV; DEP (BASC-P): The Behavior Assessment System for Children (parent report) Depression subscale; MABC-2: Movement Assessment Battery for Children-2; UF (R): Right Uncinate Fasciculus Fibre cross-section; All values are beta coefficients. Note. * *p* < 0.05 ** *p* < 0.01, *** *p* < 0.001.

Results of the mediation analysis looking at the effect of childhood MABC-2 scores on the relationship between FC of the right UF during childhood and BASC-P depression scores during adolescence are presented in Fig. 7. As Fig. 7 illustrates, results demonstrated a non-significant indirect effect (*β* = 0.03, *p* = 0.34), suggesting that MABC-2 scores did not mediate the significant association between WM during childhood and depression scores on the BASC-P during adolescence. Further model details can be accessed in Section F of the Supplementary Materials (S7).

### 2.7 Effect of right UF FC on the relationship between motor competency and parent report depression

**Fig. 8:**
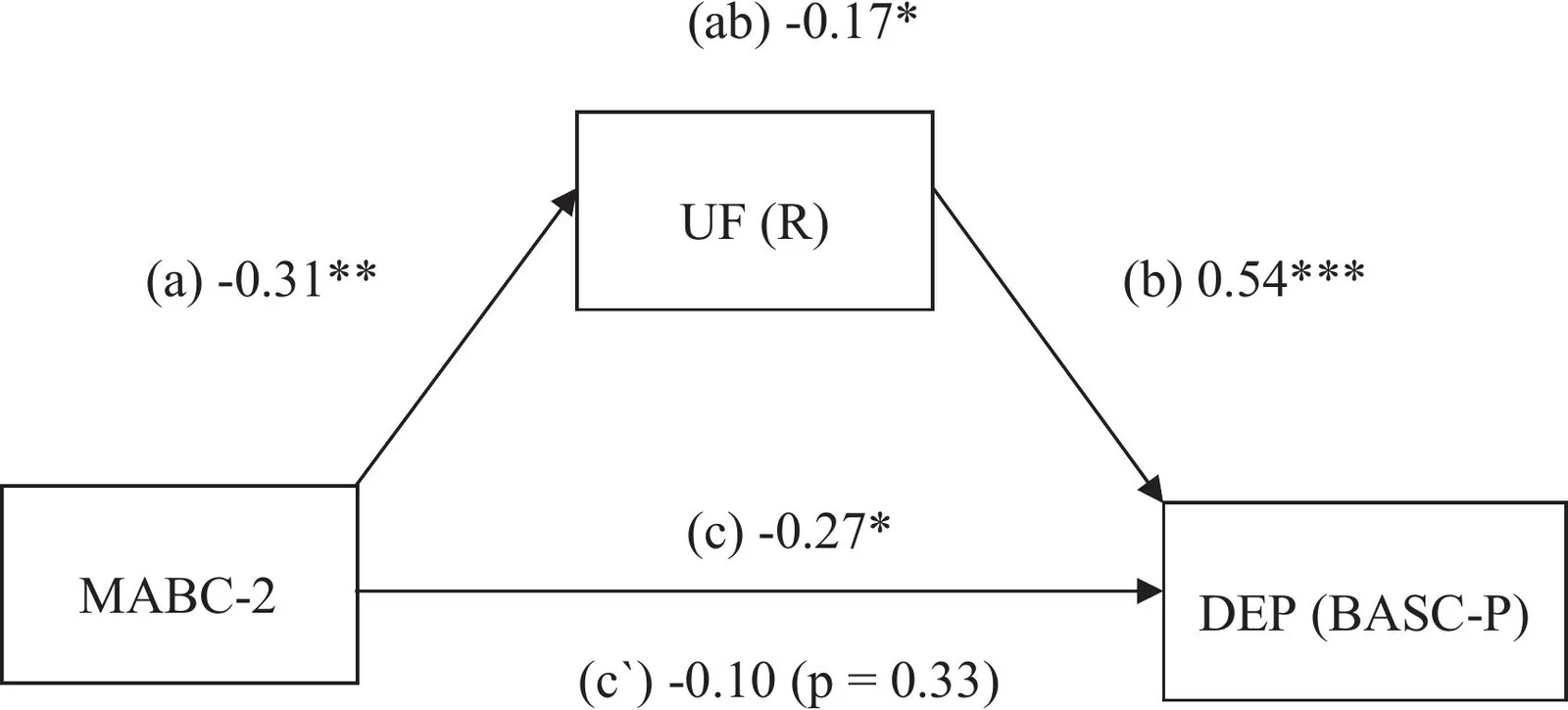
Mediation model depicting mediation of right UF FC on the relationship between childhood MABC-2 at baseline and BASC-P Depression scores during adolescence. Note: (a): IV-mediator; (b) mediator-DV; (c’) direct effect of IV-DV; (c) total effect of IV-mediator-DV; (ab) indirect effect of mediator on the IV-DV; DEP (BASC-P): The Behavior Assessment System for Children (parent report) Depression subscale; MABC-2: Movement Assessment Battery for Children-2; UF (R): Right Uncinate Fasciculus Fibre cross-section; All values are beta coefficients. Note. * *p* < 0.05 ** *p* < 0.01, *** *p* < 0.001.

Results of the mediation analysis looking at the effect of FC of the right UF during childhood on the relationship between childhood MABC-2 scores and BASC-P depression scores during adolescence are presented in Fig. 8. As Fig. 8 illustrates, results demonstrated a significant indirect effect (*β* = −0.17, *p* = 0.02), suggesting that white matter macrostructure within the right UF mediated the association between motor competency scores during childhood and depression scores on the BASC-P during adolescence. Further model details can be accessed in Section F of the Supplementary Materials (S8).

## 3 Discussion

The present study aimed to investigate whether childhood white matter organization in those with and without ADHD predicted the severity of internalizing problems in adolescence. As expected, we found qualified evidence that greater childhood FC within fronto-limbic white matter networks predicted adolescent internalizing problems. Indeed, childhood FC of the UF was a significant predictor of parent and child-rated depression scores in adolescence, while childhood FC of the bilaterial ILF was a significant predictor of parent report depression scores in adolescence. These effects appeared to be consistent across groups, and no group differences in FC were reported for those childhood white matter regions that were found to be associated with adolescent depressive symptoms. Finally, we failed to find evidence that motor ability mediated the observed relationship between white matter macrostructure within the UF of ILF and depressive symptoms. However, when we considered childhood motor ability as a predictor of adolescent depressive scores (an effect that we found to be significant, for parent reported effects), this effect was mediated by childhood FC within the UF. This work suggests that low motor function in children with and without ADHD may act as a developmental marker for emotional and behavioral dysregulation in adolescence, an effect partly explained by childhood white matter organization within fronto-limbic circuitry.

Similarly to earlier accounts (e.g. Becker and Fogleman, 2020), we observed that adolescents with ADHD presented with increased internalizing problems relative to TD controls. Specifically, adolescents with ADHD presented with greater parent report depressive symptoms (as measured by the BASC-P), relative to their TD peers. It is interesting to note that no such group differences were observed for self-report measures of internalizing problems. This finding is in-keeping with a strong body of evidence that shows that parents often report more severe symptoms of psychological distress than children and adolescents with ADHD (Fraser et al., 2018). While it is difficult to discern why these discrepancies exist, it has been suggested that children with ADHD may under-estimate the severity of their condition, though it is similarly possible that parents are more sensitive to internalizing symptoms in their children (see Fraser et al., 2018 for a discussion). Nonetheless, we note that the pattern of depressive symptoms observed in our sample of adolescents with ADHD is in-keeping with earlier accounts.

We also observed that children with ADHD presented with significant poorer motor skills than TD controls. This is shown by reduced MABC-2 scores in our sample of children with ADHD, relative to TD control children. This finding is further in-keeping with earlier accounts of the profile of motor skill in children with ADHD, where motor difficulties are commonly reported (Goulardins et al., 2017). As noted, our findings of lower motor competence in our children with ADHD are concerning, given evidence that children with ADHD who present with motor difficulties are at increased risk for developing internalizing symptoms later in life (Missiuna et al., 2014).

We commenced the present study with two alternative (though not entirely distinct) models: one being that white matter organization may precede poor motor outcomes, and that poor motor functioning then predisposes children to internalizing problems later in life (model a); and motor difficulties may be a marker for atypical white matter organization within fronto-limbic circuitry, which then contribute to difficulties with emotional and behavioral regulation (model b). These hypotheses are discussed next, in the context of our broader findings and the literature.

As expected, we found partial evidence that childhood white matter organization in fronto-limbic white matter networks predicted adolescent internalizing problems in youth with and without ADHD. Specifically, we observed that FC of the UF was a significant predictor of parent and child-rated depression scores in adolescence, even after co-varying for childhood depression levels. Childhood FC of the bilaterial ILF was also a significant predictor of parent report depression scores in adolescence, though this effect did not hold after co-varying for childhood depression levels. This demonstrates that the observed effect of the UF as a predictor of adolescent depressive symptoms cannot be attributed to baseline levels of depressive symptoms, though it may not be the case of the effect observed in the ILF. Further, no differences in FC were observed between those with ADHD and TD controls in those regions of the UF or ILF that were shown to predict depression levels. Similarly, descriptive consideration of the slopes for those with ADHD and TD controls for the relationship between FC of the UC (or ILF) and depression levels did not provide evidence of an interaction effect- that is, the relationship between childhood macrostructure and adolescent depression levels appeared to be relatively consistent between those with and without ADHD. As such, on balance, it would appear that the predictive relationship between childhood macrostructure of the UF and adolescent depression levels is consistent regardless of developmental status (ie ADHD vs TD control). These results are consistent with broader developmental literature suggesting that fronto-limbic white matter systems are critical for emotional regulation, and may be predictive of the severity of internalizing problems in children and adolescents (Ali et al., 2019; Andre et al., 2020; Chahal et al., 2021). Still, our findings are the first, to our knowledge, to show that macrostructure of the fronto-limbic pathways in childhood might predict adolescent internalizing problems in children with and without ADHD.

We also observed that motor ability in children with and without ADHD was associated with adolescent depression levels, as reported by parents. Here, lower childhood motor ability was significantly correlated with greater depressive symptoms in adolescence. This finding supports a strong body of evidence demonstrating that children with and without ADHD who present with low motor ability are at increased risk for presenting with internalizing symptoms in adolescence (Mancini et al., 2019; Missiuna et al., 2014). In doing so, our work contributes to a growing body of evidence speaking to long term impact of reduced motor competence on emotional well-being, regardless of developmental status (ala the ESH- Mancini et al., 2019). Our work here extends these earlier accounts by demonstrating that the predictive relationship between childhood motor ability and parent-report internalizing problems (specifically, depression level) may be mediated by childhood white matter organization within the UF.

With respect to the two alternative models that we aimed to test, we did not find evidence that motor ability mediated the relationship between childhood white matter of fronto-limbic circuitry and adolescent depression levels. Thus, our data did not support *model a,* which proposed that white matter organization may precede poor motor outcomes, and that poor motor functioning then predisposes children to internalizing problems later in life. However, our data did appear to support *model b*, which proposed that motor difficulties may be a marker for atypical white matter organization within fronto-limbic circuitry, which may then contribute to difficulties with emotional and behavioral regulation. Indeed, the only mediation model that we observed to be significant suggested that childhood white matter within the UF mediated the longitudinal relationship between childhood motor ability and adolescent depressive symptoms. However, we acknowledge that since motor ability and UF macrostructure were both measured at Time 1, we are unable to confirm a directional relationship between these two variables (ie. that motor competence preceded white matter organization within the UF). Instead, our findings show that the relationship between childhood motor ability and adolescent depressive symptoms are, at least partly, explained by macrostructure within the UF at childhood.

Our findings signpost the importance of considering motor outcomes in childhood ADHD, echoing recent suggestions that assessment of motor competence in childhood with ADHD be considered at presentation, and motor difficulties be directly intervened upon (Blank et al., 2019). To that latter point, by showing that childhood motor outcomes may offer a marker for atypical white matter organization and the subsequent development of internalizing problems, addressing childhood motor difficulties directly in those with (and without ADHD) may offer a cost effective adjunct to traditional treatments for improving psychological outcomes (particularly depressive symptoms) in adolescence. That is, improving motor competence in childhood may offer a ‘backdoor’ to improved psychological outcomes, and/or guard against the emergence of psychological difficulties in adolescence. There is already some evidence from earlier work that physical activity interventions in school-age children may improve internalizing symptoms (e.g. Andermo et al., 2020 for a review). Still, the majority of these prior studies demonstrate the effect across a shorter time-scale than childhood through to adolescence, and target physical activity rather than motor competence per se. Given the general health benefits of motor skill training (and the associated physical activity), future work should consider the benefits of motor skill training in childhood on reducing the severity of internalizing problems in later life in children with and without ADHD.

## 4 Conclusions

The present study aimed to investigate whether childhood white matter organization in children with and without ADHD predicted the severity of internalizing problems in adolescence. We found qualified evidence that greater childhood FC within fronto-limbic white matter networks predicted adolescent internalizing problems, effects that appeared to hold irrespective of diagnostic status (ie ADHD vs control). When we considered childhood motor ability as a predictor of adolescent depressive scores, this effect was found to be partly explained by childhood FC within the UF. Our work suggests that low motor function in children with and without ADHD may act as a marker for emotional and behavioral dysregulation in adolescence, with this effect partly explained by childhood white matter organization within fronto-limbic circuitry.

## Funding Acknowledgement

This work was funded by 10.13039/100000002National Institutes of Health (NIH), Grant/Award Numbers: K23-MH101322, P50HD103538, R01-MH078160, R01-MH085328, R03-MH119457, K23-MH107734; 10.13039/100012107Waterloo Foundation, Grant/Award Number: 2013–4545; Brain and Behavior Foundation NARSAD Young Investigator's Award; Eunice Kennedy Shriver National Institute of Child Health and Human Development. Grant Numbers: 1S10OD021648, P50HD103538.

## CRediT authorship contribution statement

**C. Hyde:** Writing – original draft, Funding acquisition, Formal analysis, Conceptualization. **I. Fuelscher:** Writing – review & editing, Funding acquisition, Formal analysis, Conceptualization. **K.S. Rosch:** Writing – review & editing, Investigation, Funding acquisition, Formal analysis, Data curation, Conceptualization. **K.E. Seymour:** Writing – review & editing, Investigation, Funding acquisition, Formal analysis, Data curation, Conceptualization. **D. Crocetti:** Writing – review & editing, Project administration, Investigation, Funding acquisition, Formal analysis, Data curation, Conceptualization. **T. Silk:** Writing – review & editing, Funding acquisition, Conceptualization. **M. Singh:** Writing – review & editing, Formal analysis. **S.H. Mostofsky:** Writing – review & editing, Investigation, Funding acquisition, Formal analysis, Data curation, Conceptualization.

## Disclaimer

Dr. Seymour currently works at the National Institute on Drug Abuse; work on this manuscript was supported while Dr. Seymour worked at Johns Hopkins/Kennedy Krieger Institute. The views and opinions expressed in this manuscript are those of the authors only and do not necessarily represent the views, official policy or position of the U.S. Department of Health and Human Services or any of its affiliated institutions or agencies.

## Data Availability

Data will be made available on request.
