# Supplementary material for: Childhood white matter organization predicts adolescent internalizing problems among youth with and without ADHD
Source: Neuroimage Clin. 2026 Sep 1;51:104053. doi: 10.1016/j.nicl.2026.104053 (PMC13571588; doi:10.1016/j.nicl.2026.104053)
Supplement: Supplementary file 1 [file mmc1.docx]

**Appendix A – Supplementary Material**

**For**

***Childhood white matter organization predicts adolescent internalizing problems among youth with and without ADHD***

**Supplementary Section A – Impact of motion**

***Framewise Displacement***

Following comparable work adopting FBA in children and adolescents (e.g., Genc et al., 2018), individual differences in head motion were estimated in each participant’s diffusion sequence (excluding b0 volumes) using mean framewise displacement (FWD) in FSL (Power et al., 2012). Further, we examined group differences in FWD between ADHD and non-ADHD groups. As the assumption of normality was violated (Shapiro Wilks, p <.05), a Mann Whitney *U* was conducted. Results of the test demonstrated no significant group differences in FWD between ADHD and non-ADHD children (*U* = 670.00, *p* = 0.61, *r* = 0.07). Further, to assess the potential impact of head motion on our results, we conducted a series of Pearson’s partial correlations (controlling for age at T1 and sex) to examine if FWD was associated with FC in the bilateral ILF, SLF, and UF. As shown in Table S1, we found no significant correlations between FWD and all tracts of interest with respect to FC.

**Table S1.**

Partial correlation matrix (Pearson’s *r*) looking at the association between FC of the bilateral ILF, SLF I-II and UF with mean FWD.

|  | | FWD |
| --- | --- | --- |
| Tracts | | *r* |
| ILF FC (left) | 0.01 | |
| ILF FC (right) | 0.07 | |
| SLF-I FC (left) | 0.13 | |
| SLF-I FC (right) | 0.14 | |
| SLF-II FC (left) | 0.06 | |
| SLF-II FC (right) | 0.07 | |
| SLF-III FC (left) | 0.03 | |
| SLF-III FC (right) | -0.06 | |
| UF FC (left) | 0.08 | |
| UF FC (right) | 0.04 | |

All coefficients are *p* > 0.05; Controlling for Age (T1) & Sex.

*Note*: ILF FC (left): Left Inferior Longitudinal Fasciculus Fibre cross-section; ILF FC (right): Right Inferior Longitudinal Fasciculus Fibre cross-section; SLF-I FC (left) Left Superior Longitudinal Fasciculus I Fibre cross-section; SLF-I FC (right) Right Superior Longitudinal Fasciculus I Fibre cross-section; SLF-II FC (left) Left Superior Longitudinal Fasciculus II Fibre cross-section; SLF-II FC (right) Right Superior Longitudinal Fasciculus II Fibre cross-section; SLF-III FC (left) Left Superior Longitudinal Fasciculus III Fibre cross-section; SLF-III FC (right) Right Superior Longitudinal Fasciculus III Fibre cross-section; UF FC (left): Left Uncinate Fasciculus Fibre cross-section; UF FC (right): Right Uncinate Fasciculus Fibre cross-section.

***The impact of FWD on the observed effects between FC and internalizing problems***

The correlation between mean FC in those regions where an association was observed between FC and internalizing symptoms where FWD was and was not controlled for were as follows:

|  | Tract | Correlation |
| --- | --- | --- |
| Figure 3 | BASC-P DEP UF Left | *r* = .995, p < .001 |
|  | BASC-P DEP UF Right | *r* = .971, p < .001 |
|  | BASC-P DEP ILF Left | *r* = .962, p < .001 |
|  | BASC-P DEP ILF Right | *r* = .999, p < .001 |
| Figure 4 | BASC-C DEP UF Left | *r* = .996, p < .001 |
|  | BASC-C DEP UF Right | *r* = .872, p < .001 |

**Supplementary Materials Section B – Predicting adolescent internalizing problems from childhood white matter organization (inclusion of R and p values for each group individually).**

Figure S1. Tract specific streamlines showing a significant positive association between FC of the (A) bilateral UF and (B) bilateral ILF during childhood with scores on the parent report BASCP Depression subscale in adolescence. Scatterplots show the linear relationship between FC of the bilateral UF and ILF for each group.

Figure S2. Tract specific streamlines showing a significant positive association between FC of the (A) bilateral UF during childhood with scores on the self-report BASC-C Depression subscale during adolescence and (B) left UF during childhood with scores on the self report BASCC Anxiety subscale during adolescence. Scatterplots show the linear relationship between FC of and Internalizing scores for each group

**Supplementary Materials Section C – CFE analysis controlling for CBCL scores at Time-point 1**

***Part A - Parent report BASC***

Figure S3. Tract specific streamlines showing a positive association between FC of the bilateral UF with scores on the parent report BASC Depression subscale after covarying for CBCL scores at T1. Blue streamlines represent fixels significant at *p* < 0.05 _FWE_, purple streamlines represent fixels demonstrating a trend towards significance (*p* < 0.1 _FWE_) Scatterplots show the linear relationship between BASC Depression scores and FC of significant fixels within the (a) right and (b) left UF for each group.

***Part B – Child/Self report BASC***

Figure S4. Tract specific streamlines showing a positive association between FC of the left UF with scores on the self-report BASC Depression subscale after covarying for CBCL scores at T1. Blue streamlines represent fixels significant at *p* < 0.05 _FWE_, purple streamlines represent fixels demonstrating a trend towards significance (*p* < 0.1 _FWE_) Scatterplots show the linear relationship between BASC Depression scores and FC of significant fixels within the left UF for each group.

**Supplementary Materials Section D – Linear-mixed modelling analysis**

**Table S3.**

Fixed effects Omnibus tests for the linear mixed model looking at group differences in FC on for the BASCP CFE analysis.

| Fixed effects | *F* | *p* |
| --- | --- | --- |
| Tract | 19.22 | <.001 |
| Group | 2.50 | 0.12 |
| TCV | 86.74 | <.001 |
| Age (T1) | 6.07 | 0.02 |
| Sex | 0.06 | 0.81 |
| Tract*Group | 0.15 | 0.93 |

**Table S4.**

Fixed effects Omnibus tests for the linear mixed model looking at group differences in FC on for the BASCC CFE analysis.

| Fixed effects | *F* | *p* |
| --- | --- | --- |
| Tract | 52.96 | <.001 |
| Group | 0.55 | 0.46 |
| TCV | 48.59 | <.001 |
| Age (T1) | 0.63 | 0.43 |
| Sex | 1.03 | 0.32 |
| Tract*Group | 1.38 | 0.26 |

**Supplementary Materials Section E – Sex differences on behavioral measures**

***Sex differences on behavioral measures***

Further subgroup analyses examining sex differences on the parent report BASCP demonstrated significant interaction effects between diagnostic status and sex on depression, *F* (1, 72) = 6.61, *p* = 0.01, ηp^2^ = 0.08, and somatization, *F* (1, 72) = 4.80, *p* = 0.03, ηp^2^ = 0.06. Tukey’s post-hoc analyses found that females without ADHD had lower depression (*est* = -14.31; *p_Tukey_* < 0.001) and somatization scores (*est* = -11.93; *p_Tukey_* < 0.05) than females with ADHD. ADHD diagnosed females also scored higher on depression compared to males with (*est* = 3.06; *p_Tukey_* < 0.05) and without ADHD (est = 3.96; *p_Tukey_* = 0.001). Given that there were no significant group differences between ADHD and non-ADHD adolescents on all subscales of the self report BASCC, we did not run further sex-specific subgroup analyses.

**Supplementary Materials Section F – Mediation Analysis**

***Part A -Assumption testing***

**Table S4.**

Partial correlations (Pearson’s *r*) between FC, MABC-2, and BASCP Depression

|  | BASCP DEP | MABC-2 |
| --- | --- | --- |
| BASCP DEP | - |  |
| MABC-2 | -0.26* | - |
| ILF FC (left) | 0.33** | -0.08 |
| ILF FC (right) | 0.41*** | -0.25* |
| UF FC (left) | 0.40*** | -0.16 |
| UF FC (right) | 0.55*** | -0.30* |
|  |  |  |

* *p* < 0.05 ** *p* < 0.01, *** *p* < 0.001; controlling for Age (T1), Sex & Total Cranial Volume (TCV).

*Note*: BASCP DEP: The Behavior Assessment System for Children (parent report) Depression subscale; MABC-2: Movement Assessment Battery for Children-2; ILF FC (left): Left Inferior Longitudinal Fasciculus Fibre cross-section; ILF FC (right): Right Inferior Longitudinal Fasciculus Fibre cross-section; UF FC (left): Left Uncinate Fasciculus Fibre cross-section; UF FC (right): Right Uncinate Fasciculus Fibre cross-section.

**Table S5.**

Partial correlations (Pearson’s *r*) between FC, MABC-2, and BASCC Depression & Anxiety.

|  | BASCC DEP | MABC-2 |
| --- | --- | --- |
| BASCC DEP | - |  |
| MABC-2 | -0.01 | - |
| UF FC (left) | 0.47*** | -0.12 |
| UF FC (right) | 0.38** | -0.22 |
|  | BASCC ANX | MABC-2 |
| BASCC ANX | - |  |
| MABC-2 | -0.10 | - |
| UF FC (left) | 0.27* | -0.02 |
|  |  |  |
|  |  |  |

* *p* < 0.05 ** *p* < 0.01, *** *p* < 0.001; controlling for Age (T1) & Sex & Total Cranial Volume (TCV).

*Note*: BASCC DEP: The Behavior Assessment System for Children (child report) Depression subscale; MABC-2: Movement Assessment Battery for Children-2; BASCC ANX: The Behavior Assessment System for Children (child report) Anxiety subscale; UF FC (left): Left Uncinate Fasciculus Fibre cross-section; UF FC (right): Right Uncinate Fasciculus Fibre cross-section.

**Part B – Simple Mediation**

**Table S6.**

Mediation table showing the relationship between right ILF FC (right) (IV), MABC-2 (Mediator) & BASCP DEP (DV)

| Path | Estimate | SE | *β* | *p* |
| --- | --- | --- | --- | --- |
| ab | 0.04 | 0.03 | 0.04 | 0.21 |
| a | -0.25 | 0.11 | -0.25 | 0.03 |
| b | -0.17 | 0.11 | -0.16 | 0.13 |
| c' | 0.40 | 0.11 | 0.40 | <.001 |
| c | 0.44 | 0.11 | 0.44 | <0.01 |
| *Note*: (ab) Indirect effect of mediator on the IV-DV relationship; (a) IV-mediator; (b) mediator-DV; (c’) direct effect of IV-DV; (c) total effect of IV-mediator-DV.  *Note*: BASCP DEP: The Behavior Assessment System for Children (parent report) Depression subscale; MABC-2: Movement Assessment Battery for Children-2; ILF FC (right): Right Inferior Longitudinal Fasciculus Fibre cross-section  *Note*: Confidence intervals computed with method: Standard (Delta method) | | | | |
| *Note*: Betas are completely standardized effect sizes | | | | |
| *Note*: Residualized scores used for FC and BASCP Depression scores | | | | |

**Table S7.**

Mediation table showing the relationship between right UF FC (right) (IV), MABC-2 (Mediator) & BASCP DEP (DV)

| Path | Estimate | SE | *β* | *p* |
| --- | --- | --- | --- | --- |
| ab | 0.03 | 0.03 | 0.03 | 0.36 |
| a | -0.30 | 0.11 | -0.31 | 0.006 |
| b | -0.10 | 0.10 | -0.10 | 0.33 |
| c' | 0.54 | 0.10 | 0.54 | < .001 |
| c | 0.57 | 0.10 | 0.57 | < .001 |
| *Note*: (ab) Indirect effect of mediator on the IV-DV relationship; (a) IV-mediator; (b) mediator-DV; (c’) direct effect of IV-DV; (c) total effect of IV-mediator-DV.  *Note*: BASCP DEP: The Behavior Assessment System for Children (parent report) Depression subscale; MABC-2: Movement Assessment Battery for Children-2; UF FC (right): Right Uncinate Fasciculus Fibre cross-section.  *Note*. Confidence intervals computed with method: Standard (Delta method) | | | | |
| *Note*: Betas are completely standardized effect sizes | | | | |
| *Note*: Residualized scores used for FC and BASCP Depression scores | | | | |

**Table S8.**

Mediation table showing the relationship between MABC-2 (IV), right UF FC (mediator) & BASCP DEP (DV)

| Path | Estimate | SE | *β* | *p* |
| --- | --- | --- | --- | --- |
| ab | -0.17 | 0.07 | -0.17 | 0.02 |
| a | -0.31 | 0.11 | -0.31 | 0.006 |
| b | 0.54 | 0.10 | 0.54 | < .001 |
| c' | -0.10 | 0.10 | -0.10 | 0.33 |
| c | -0.27 | 0.12 | -0.26 | 0.02 |
| *Note*: (ab) Indirect effect of mediator on the IV-DV relationship; (a) IV-mediator; (b) mediator-DV; (c’) direct effect of IV-DV; (c) total effect of IV-mediator-DV.  *Note*: MABC-2: Movement Assessment Battery for Children-2; BASCP DEP: The Behavior Assessment System for Children (parent report) Depression subscale; UF FC (right): Right Uncinate Fasciculus Fibre cross-section.  *Note*. Confidence intervals computed with method: Standard (Delta method) | | | | |
| *Note*: Betas are completely standardized effect sizes | | | | |
| *Note*: Residualized scores used for FC and BASCP Depression scores | | | | |

**References**

Genc, S., Smith, R. E., Malpas, C. B., Anderson, V., Nicholson, J. M., Efron, D., Sciberras, E., Seal, M. L., & Silk, T. J. (2018). Development of white matter fibre density and morphology over childhood: A longitudinal fixel-based analysis. *NeuroImage*, *183*, 666–676. APA PsycInfo. https://doi.org/10.1016/j.neuroimage.2018.08.043

Power, J. D., Barnes, K. A., Snyder, A. Z., Schlaggar, B. L., & Petersen, S. E. (2012). Spurious but systematic correlations in functional connectivity MRI networks arise from subject motion. *NeuroImage*, *59*(3), 2142–2154. APA PsycInfo. https://doi.org/10.1016/j.neuroimage.2011.10.018
